# Physical Properties of Lens Membranes in Animals with Different Lifespans

**DOI:** 10.3390/biom15060851

**Published:** 2025-06-10

**Authors:** Marija Raguz, Witold Karol Subczynski

**Affiliations:** 1 Department of Medical Physics and Biophysics, University of Split School of Medicine, 21000 Split, Croatia; 2 Department of Biophysics, Medical College on Wisconsin, Milwaukee, WI 53226, USA

**Keywords:** eye lens, phospholipids, cholesterol, protection mechanisms, lifespan

## Abstract

The lipid composition of eye lens fiber cell membranes varies among species, and these differences increase as a function of lifespan. However, the way in which the lipids of the fiber cell membranes are organized in different animals has not yet been studied in detail. This study compares how the structure, properties, and organization of lipids change during the lifespan of the mouse, pig, and human. These species were chosen because of the wide range of lifespans and significant differences in lipid composition. Models were made of phospholipid compositions resembling those of the lens fiber cell membranes of a mouse, pig, and human aged of 3, 23, and 70 years, respectively. To separate the effects of phospholipids and cholesterol, membranes were investigated for samples without cholesterol, with a cholesterol content close to the cholesterol saturation limits, and with a cholesterol content close to the cholesterol solubility thresholds. Membrane physical properties were obtained using EPR spin labeling methods. No significant differences in the physical properties of the membranes of any of the models were detected for membranes without and those in the presence of a saturating cholesterol concentration. Thus, not only cholesterol but also the appropriate phospholipid composition is a significant factor in maintaining eye lens homeostasis.

## 1. Introduction

The changes in phospholipid (PL) composition and the cholesterol (Chol) content in eye lens fiber cell membranes of different animals as a function of animal lifespan have been investigated, and results have been published in many papers [1,2,3,4,5,6,7,8]. Not too much attention has been paid, however, to the differences in the organization of lipids and physical properties of the lipid bilayer portion of fiber cell membranes among animals of different lifespans. It is commonly accepted that Chol is the major lipid that regulates membrane fluidity [9,10,11,12,13,14] or, more accurately, the physical properties of the membranes [10,15,16,17,18]. Also, the effects of PL composition on the physical properties of membranes cannot be neglected. In the absence of Chol, the membrane’s physical properties can be very different for lipid bilayers made of different PLs [15,16,17,18]. The fiber cell plasma membrane separates the cell’s cytoplasm from its external environment, and its lipid bilayer portion determines bulk membrane properties, including diffusion barriers [10,19,20]. These barriers should be very high to block the nonspecific permeation of small polar and nonpolar molecules, allowing membrane proteins to effectively control transport and communication between the cell cytoplasm and the extracellular environment. In fiber cell plasma membranes, the saturating Chol content serves this purpose very well because Chol raises the hydrophobicity barrier in the membrane center for the permeation of polar molecules and the rigidity barrier for the permeation of nonpolar molecules near the membrane surface [21,22,23]. The physical properties of the lipid bilayer portion of biological membranes are significant because they are involved in the regulation of the functional activity of integral membrane proteins immersed in these bilayers [12,24,25]. These regulatory properties were also indicated for fiber cell membranes and the activity of aquaporins, the major integral proteins of these membranes [26,27,28,29,30]. All these factors indicate that the investigation of the physical properties of fiber cell membranes for animals of different lifespans, and thus membranes with different lipid compositions, is needed to better understand the relationships between changes in the lipid composition and changes in the structure, properties, and organization of lipids in the lipid bilayer portion of fiber cell membranes. In this paper, we look at how all these changes are related to the functioning of eye lenses in animals with different lifespans.

For the above-mentioned purpose, we chose to investigate three representative animals, the mouse, the pig, and the human, with lifespans of 3, 23, to 70 years, respectively. These species also differ significantly in phospholipid composition, with a phosphatidylcholine content that decreases as a function of lifespan (46% for the mouse, 35% for the pig, and 11% for the human) and an increased sphingolipid content (15% for the mouse, 29% for the pig, and 66% for the human) [4]. This wide spectrum of compositional differences should help to explain changes in membrane organization as a function of lifespan.

Our goal was to determine the properties of the lipid bilayer portion of fiber cell plasma membranes using EPR spin labeling methods. However, the profiles of certain membrane properties obtained for the lipid bilayer portion of intact membranes are contaminated by boundary lipids, which significantly change the measured values and the shape of the profiles [21,23]. More appropriate profiles can be obtained for lens lipid membranes (LLMs) prepared from total lipid extracts from the appropriate fiber cell membranes with a fixed PL composition and Chol content. Such work has already been conducted for human LLMs [22,23] and for porcine LLMs [21,31]. To understand the major mechanisms that determine the physical properties of the lipid bilayer portion of intact membranes and separate the effects of PL compositions and Chol, we created three models for each animal: membranes without cholesterol, membranes with a cholesterol content close to the cholesterol saturation limits, and membranes with a cholesterol content close to the cholesterol solubility thresholds. Membranes with PL compositions close to those in the membranes of the investigated animals, but with saturating and oversaturating Chol contents, form more authentic models. However, in many of our papers, we have shown that the saturating and oversaturating Chol contents in PL membranes ensure the stability of the structure, properties, and organization of lipids in these membranes, independent of changes in the membrane’s PL composition [20,21,22,23]. Membranes without Chol, but with PL compositions close to those in the membranes of the investigated animals, are not models for fiber cell membranes but were investigated to better understand the effects of Chol on membrane properties. Although the profiles of membrane properties were investigated for the individual PLs [10,17,18,32], detailed properties of certain PL mixtures were not investigated. It is significant to show how membranes comprising PL mixtures will vary membrane properties.

## 2. Materials and Methods

### 2.1. Materials

All PLs [phosphatidylcholine (PC), phosphatidylethanolamine (PE), phosphatidylserine (PS), and egg chicken sphingomyelin (SM)], Chol, and PL spin labels [1-palmitoyl-2-(n-doxylstearoyl) phosphatidylcholine (n-PC, where n = 5, 7, 10, 12, 14, or 16) and tempocholine-1-palmitoyl-2-oleoylphosphatidic acid ester (T-PC)] were obtained from Avanti Polar Lipids (Alabaster, AL, USA). Ninedoxylstearic acid spin labels (9-SASL), cholestane spin label (CSL), and androstane spin label (ASL) were purchased from Molecular Probes (Eugene, OR, USA). Other chemicals (of at least reagent grade) were purchased from Sigma-Aldrich (St Louis, MO, USA).

### 2.2. Preparation of Model Membranes

The membranes used in this study are multilamellar dispersions of appropriate PL mixtures without Chol, with a Chol concentration saturating the PL bilayers, and with a Chol content exceeding the saturation limits [i.e., when pure Chol bilayer domains (CBDs) are already present within the PL bilayers]. Samples containing 1 mol% spin label were prepared using the film deposition method given in [33,34]. Chloroform solutions of PLs, Chol, and spin label were mixed to obtain the desired mixing ratios. Chloroform was evaporated with a stream of nitrogen in order to deposit a uniform film of lipid over the bottom of the tube. The lipid film was thoroughly dried under reduced pressure (0.1 mm Hg) for 12 h. A buffer solution (0.2 mL 10 mM PIPES and 150 mM NaCl, pH 7.0) was added to and vigorously mixed with the dried lipids at 50 °C. The buffer used for samples with 9-SASL was 0.1 M borate at pH 9.5. A rather high pH was chosen in this case to ensure that all 9-SASL carboxyl groups were ionized in the PL membranes [35,36].

#### 2.2.1. PL Composition

For our investigations, we took the total PL composition provided in [4] for four majors PLs—PC, PE, PS, and SM—of the eye lens fiber cell membranes of the mouse, pig, and human. The PL compositions for the three models are as follows: For the mouse model, this composition included 46 mol% of PC, 17 mol% of PE, 17 mol% of PS, and 15 mol% of SM. For the pig model, this composition included 35 mol% of PC, 12 mol% of PE, 21 mol% of PS, and 29 mol% of SM. For the human model, this composition included 11 mol% of PC, 15 mol% of PE, 15 mol% of PS, and 66 mol% of SM. In [4], all of the sphingolipid mixtures also included dihydrosphingomyelin (DHSM). In our experiments, we used only SM as a total sphingolipid amount. Information about the composition of acyl chains of certain PLs is not available; therefore, we chose the biological membrane composition of palmitic (P) and oleic (O) fatty acids (POPC, POPE, and POPS), which are more commonly used for modeling. We justified our decision by the fact that the eye lens fiber cell membranes of young animals consist of ~33% palmitate and 33% oleate [4]. The decreases in the relative abundance of oleate found in deeper regions of the lens are consistent with the observed disappearance of PE and PS in the inner fractions. The concomitant increase in palmitate and nervonate is due to the relative increase in SM [37,38].

#### 2.2.2. Chol Content

To observe the effects of Chol on the membranes of the investigated models, we chose two distinct Chol/PL mixing ratios for all PLs used to form the PL mixtures described in Section 2.2.1. We used Chol/PL mixing ratios of the Chol saturation limits and the Chol solubility thresholds (CSTs) at which CBDs and Chol crystals start to form, respectively. Data from the literature provides values for CSTs [39,40,41,42,43,44]. However, the obtained values vary and strongly depend on the methods with which the investigated PL bilayers were formed [39,40,43,44]. The artefactual formation of Chol crystals during membrane preparations using the film deposition method caused a decrease in the Chol/PL ratio in the bilayer as compared with the Chol/PL mixing ratio during bilayer preparations; this was a major problem. Therefore, we made our own evaluations of the Chol/PL mixing ratios at which membranes are saturated with Chol (approaching the Chol solubility limits) and oversaturated with Chol (CBDs were already formed in these membranes). These procedures are described in Section 3.3. Finally, we used a Chol/PL mixing ratio slightly lower than 1.0 for the mouse model, slightly lower than 1.2 for the pig model, and slightly lower than 1.6 for the human model. To ensure the presence of CBDs in the investigated membrane models, the Chol/PL mixing ratios were 2.1 for the mouse model, 2.4 for the pig model, and 3.2 for the human model.

### 2.3. Conventional and Saturation-Recovery Electron Paramagnetic Resonance

For electron paramagnetic resonance (EPR) measurements, spin-labeled membrane suspensions were centrifuged for a short time and a loose pellet was transferred to a capillary made of gas-permeable methylpentene polymer (TPX). To further increase the signal-to-noise ratio, the sample in the TPX capillary was centrifuged as described in [45]. Conventional EPR measurements were performed on a Bruker EMX spectrometer equipped with a Super High Q cavity and temperature-control accessories. All samples were thoroughly deoxygenated, yielding correct EPR line shapes. Conventional EPR measurements taken at the X-band and performed at 37 °C were used to measure the transmembrane profiles of the order parameter of hydrocarbon chains [46] (see Supporting Materials and Figure S1 for a detailed explanation). The EPR spectra measured for samples frozen at −165 °C were used to obtain the *z*-component of the hyperfine interaction tensor of the n-PC, 9-SASL, and T-PC, *A*_Z_, and construct the transmembrane profiles of hydrophobicity [15,17] (see Supporting Materials and Figure S2 for a detailed explanation).

The saturation-recovery (SR) EPR signal for each sample was obtained on the central line of the spin label EPR spectrum by short-pulse SR EPR at the X-band with the use of a loop-gap resonator [47] (see Supporting Materials and Figure S3 for a detailed explanation). The pump arm in this SR system is now capable of delivering a pulse width as narrow as 10 ns at a 1 W level of power to the LGR. The availability of this level of pump power ensures the saturation of the sample with the narrow pump pulse widths needed to detect the faster components present in multiexponential signals. The home-built SR spectrometer was first described in the SR review by Hyde [48]. All measurements were carried out at 37 °C for samples equilibrated with the same gas that was used for temperature control (i.e., a controlled mixture of nitrogen and dry air adjusted with flowmeters) [45]. A relatively low level of observing power (8 µW, with a loop-gap resonator delivering an *H*_1_ field of 3.6 × 10^−5^ gauss) was used for all experiments to avoid microwave power saturation, which induces artificial shortening of the apparent spin–lattice relaxation time (*T*_1_). Accumulations of the decay signals were carried out with 2048 data points on each decay. SR signals were fitted through the use of single-exponential functions (see Figure S3A):

*I*(t) = *I*_o_exp − (t*T*_1_^−1^), (1)

where *I*(t) and *I*_o_ are, respectively, the amplitudes of SR signals at time t and immediately after the end of the saturating microwave pulse. *T*_1_ is the spin–lattice relaxation time. When appropriate, the double-exponential function was used to fit the SR signals (see Figure S3B):

*I*(t) = *I*_o1_exp − (t*T*_11_^−1^) + *I*_o2_exp − (t*T*_12_^−1^), (2)

where the subscripts 1 and 2 indicate parameters coming from two different lipid environments. When a single-exponential fit was satisfactory, the uncertainties in the measurements of decay time from the fits were usually less than 0.05%, whereas the decay times determined from sample to sample (i.e., for samples prepared totally independently) were within an accuracy of ±3%. When a double-exponential fit was necessary, and satisfactory, the decay times were usually evaluated with standard deviations less than ±5 and ±10% for longer and shorter recovery time constants, respectively.

## 3. Results

The obtained profiles can be divided into those monitoring nondynamic properties (i.e., profiles of the order parameter and hydrophobicity) obtained with the conventional EPR technique and dynamic membrane properties (i.e., profiles of the spin–lattice relaxation rate and oxygen transport parameter) obtained with the SR EPR technique. Also, the obtained profiles can be grouped as those describing the properties of parent molecules or, more accurately, the properties of the fragments of these molecules to which the nitroxide moiety is rigidly attached, including profiles of the order parameter and fluidity (spin–lattice relaxation rates). Profiles of the oxygen transport parameter and hydrophobicity characterize the physicochemical properties of the microenvironment of the nitroxide moiety. We grouped our results into those describing the properties of the parent molecules, namely, PLs and Chol, and those describing oxygen diffusion and concentration across the PL bilayers and the hydrophobicity of the bilayer interior.

The profiles of the PL bilayer properties obtained with PL-analog spin labels are affected by the Chol molecules incorporated in the bilayer, but at the same time, they are not contaminated by the presence of pure CBDs in membranes. This is guaranteed (assured) by the fact that PL-analog spin labels do not partition into the pure CBDs. The presence of the CBDs ensures that the surrounding PL bilayer is saturated with Chol (i.e., that it has already reached the Chol saturation limit). Chol-analog spin labels not only allow the discrimination of CBDs immersed into the PL bilayer saturated with Chol but also provide information about PL bilayer properties sensed by Chol molecules.

### 3.1. Profiles of PL Acyl Chain Order and Fluidity

**Figure 1:**
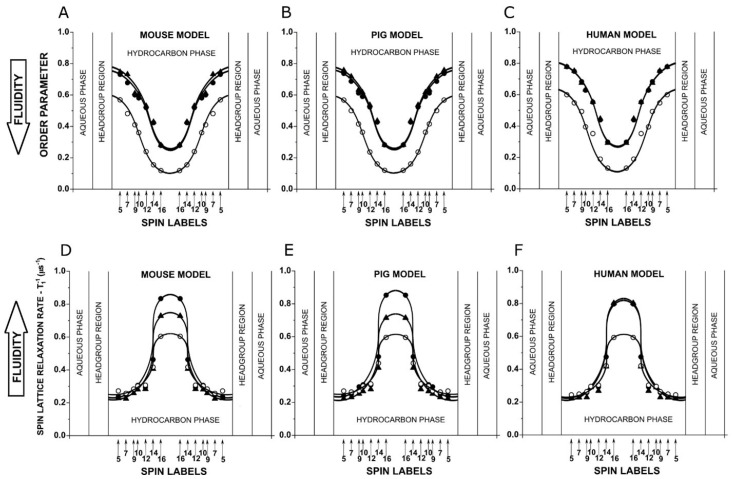
Profiles of the order parameters (**A**–**C**) and spin–lattice relaxation rates (*T*_1_^−1^) (**D**–**F**) for mouse (**A**,**D**), pig (**B**,**E**), and human (**C**,**F**) models. Profiles obtained for lipid bilayers without Chol (○), bilayers with a Chol content at the saturation limit (●) (CBDs are not yet formed), and those with a Chol content well above the Chol saturation limit (▲) (CBDs are already formed). All profiles were obtained at 37 °C with the PL-analog spin labels n-PCs (n = 5, 7, 10, 12, 14, and 16) and 9-SASL and symmetrized for both bilayer leaflets. Approximate locations of the nitroxide moieties of spin labels are indicated by arrows (see References [51,52,53] for details on how these localizations were determined). For easier comparison of membrane properties, we adjusted profiles without and with Chol by the localization of the nitroxide moieties of spin labels in both bilayers. The thicknesses of the hydrocarbon phase of the bilayer can be calculated as described in [54], from published membrane thickness data [55], the surface area of the DMPC moiety [56], and the average volume of CH_2_ groups (assuming volume of CH_3_ = 2 volumes of CH_2_) as given in [57]. Because Chol does not affect the thickness of polar headgroups [58], its effect on the hydrocarbon phase could be estimated from data on the thickness of Chol-containing lipid bilayers [55,59,60]. For a detailed description of the procedures, see [54].

Figure 1 presents the profiles of two membrane physical property parameters that describe the order and fluidity of the acyl chains of PL molecules. Both are straightforwardly connected with properties of the PLs because the nitroxide moiety (the EPR monitoring group) is rigidly attached to an acyl chain at a different position. The order parameter (Figure 1A–C) describes the angular amplitude of the local wobbling motion of the fragment of the acyl chain to which the nitroxide is attached. The cone, to which membranes confine this wobbling motion, effectively increases along the chain, and the value of the order parameter decreases. The order parameter is nondynamic because it does not contain information about time or velocity. The profiles of the spin–lattice relaxation rate, *T*_1_^−1^, depend on the rate of the spin label rotational diffusion [49,50] and thus the motion of the acyl chain fragment to which the nitroxide moiety is rigidly attached [16]. Thus, these profiles show the change in fluidity along the PL acyl chains.

To better understand membrane properties described by the order parameter and *T*_1_^−1^ profiles, we should consider that acyl chain order and fluidity result from *trans-gauche* isomerization, which takes place simultaneously at different positions along the chain and has a cumulative effect. Because of this, both profiles—in the absence and in the presence of Chol—are bell-shaped.

Based on our previous results for PL membranes saturated with Chol, we expected that the profiles of the order parameter and fluidity for membranes saturated and oversaturated with Chol will be similar (identical) for all three membrane models. We have shown in many of our papers [21,22,23] that the saturating Chol content in lipid bilayers ensures that the bulk physical properties across these membranes are consistent with and independent of PL compositions. However, similarities in profiles for membranes without Chol for mouse, pig, and human models were not expected. These membranes consist of different mixtures of fiber cell membrane PLs (see Section 2.2.1); however, the major differences between models are for PC (46 mol% for mouse, 35 mol% for pig, and 11 mol% for human models) and for SM (15 mol% for mouse, 29 mol% for pig, and 66 mol% for human models). The contribution of PE and PS differs between models within 5 mol% and 6 mol%, respectively, without any defined (specified) trend. The profiles of the physical properties of membranes made of single POPC [18,61] and single SM [17] differ significantly; however, in their mixture—together with added POPE and POPS—these substantial differences do not affect the final profiles, which we also did not expect.

The most significant part of our research is that on the effect of Chol on these profiles, because Chol is the major lipid component in eye lens fiber cell membranes, ensuring their homeostasis [10,18,20]. The profiles of the order parameter for membranes without Chol and those for membranes saturated and oversaturated with Chol differ significantly, showing the typical ordering effect of Chol on acyl chains at all depths in the membrane (Figure 1A–C). Here, we should mention that these profiles are nondynamic. Fluidity profiles (Figure 1D–F), which describe rotational motions of the appropriate segment of the acyl chain obtained for membranes without Chol and those for membranes saturated and oversaturated with Chol, are similar. In all three models, in the presence of Chol, a noticeable decrease was observed in the fluidity and in the hydrocarbon core of the membranes to the depth of C9, the depth to which the rigid structure of the Chol molecule reached into the bilayer. Also, close to the membrane center, the presence of Chol induced a noticeable increase in membrane fluidity. In single-PL membranes, such changes in membrane fluidity induced by the presence of Chol were more pronounced [17,18]. They are straightforwardly connected with the rigidifying effects of Chol to the depth of C9 and the fluidizing effect of Chol in the membrane center, at locations deeper that that reached by the Chol steroid ring [62].

### 3.2. Physicochemical Properties of the Environment of Spin Labels (OTP and Hydrophobicity)

**Figure 2:**
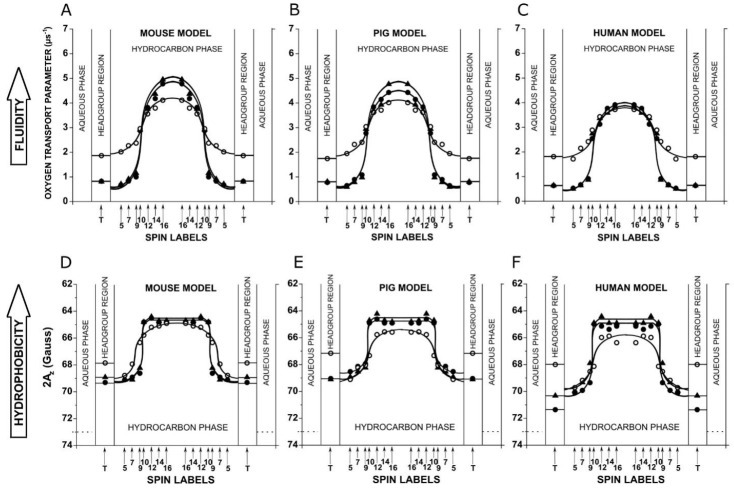
Profiles of the oxygen transport parameter (**A**–**C**) and hydrophobicity (**D**–**F**) for mouse (**A**,**D**), pig (**B**,**E**), and human (**C**,**F**) models. Symbols and explanations are the same as in the caption of Figure 1.

Figure 2 shows the profiles of two membrane physical property parameters that describe the oxygen transport parameter (OTP) and hydrophobicity sensed by nitroxide moieties of spin labels in their close vicinity. Because of this, these profiles are not affected by the cumulative effect like the profiles of the order parameter and *T*_1_^−1^. The profiles of the OTP (Figure 2A–C) describe dynamic membrane properties, namely, membrane fluidity sensed by the diffusion of a small probe molecule, molecular oxygen. The SR EPR approach allows the measurement of the collision rate between molecular oxygen and the nitroxide moiety located at a specific place in the membrane, which depends on the local oxygen concentration and the local oxygen diffusion (oxygen diffusion–concentration product). The OTP was introduced by Kusumi et al. in 1982 to measure this product [16] and is used to study the dynamic structure of model and biological membranes.

The hydrophobicity profiles for all membrane models are presented in Figure 2D–F. The conventional EPR method, which allows such profiles to be measured, is based on the fact that the polar solvents increase the *z*-component of the hyperfine interaction measured as the maximum splitting in the EPR spectrum of a spin label frozen in membrane suspension, *A*_Z_ [15,53,63]. Smaller 2*A*_Z_ values (upward changes in the profiles) indicate a more hydrophobic environment. These profiles describe the shape of the hydrophobic barrier for the nonspecific permeation of small polar molecules across the membrane.

As shown in Figure 2, in the absence of Chol, both OTP and hydrophobicity profiles are bell-shaped with a gradual increase in measured values toward the membrane center. Both profiles are very similar for all three membrane models regardless of the fact that they can be very different for single bilayers (i.e., comparing the hydrophobicity profiles for POPC [18] and SM [17]). We think that these similarities, especially for hydrophobicity profiles, are the result of the effects of acyl chain unsaturation, which significantly increases the hydrophobicity of the membrane interior [15].

As expected, the profiles of OTP and hydrophobicity measured for mouse, pig, and human models, for membranes saturated and oversaturated with Chol, are similar (Figure 2). For all three models, Chol significantly decreased OTP values in the polar headgroup region and in the hydrocarbon region up to the depth of C9, the depth to which the Chol rigid fused-ring structure is immersed (Figure 2A–C). At deeper locations, OTP values increased abruptly by about three times between C9 and C10. At locations deeper than C10, the OTP value further increased toward the membrane center with a bell-shaped profile. The reduction in the OTP in the polar headgroup region and to the depth of C9 is practically the same for all membrane models. Chol increased OTP values at the membrane center most in the mouse model, less in the pig model, and to a negligible extent in the human model. The effects of Chol on hydrophobicity profiles (Figure 2D–F) are, in some way, qualitatively like the effects of Chol on OTP profiles. A big decrease in hydrophobicity was observed in the polar headgroup region. This was smallest for the mouse model, larger for the pig model, and largest for the human model. However, in the membrane hydrocarbon region to the depth of C9, the effects of Chol are not pronounced. Similarly to OTP, membrane hydrophobicity increased abruptly between the C9 and C10 positions on the acyl chains in all three membranes. The observation of this abrupt increase in the OTP and hydrophobicity was possible because the cumulative effect does not affect these parameters, and it changes sharply within the distance of one C-C bond.

### 3.3. Membrane Properties Sensed by Chol-Analog Spin Labels

**Figure 3:**
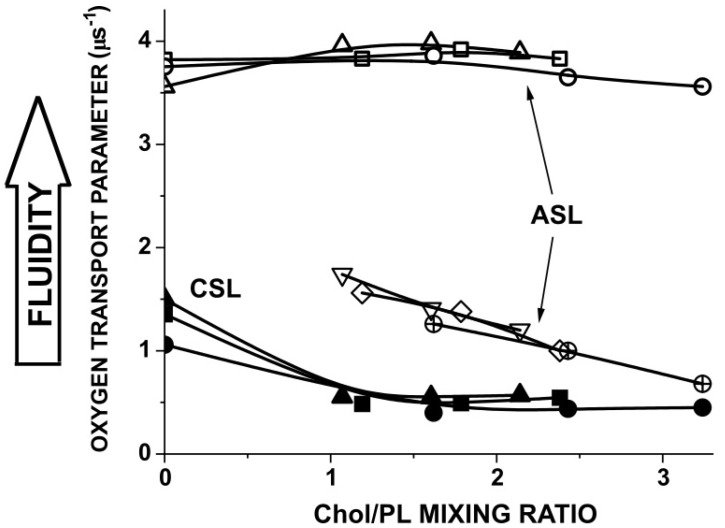
Values of the oxygen transport parameter obtained with Chol-analog spin labels ASL (at the membrane center) and CSL (in the polar headgroup region) presented as a function of the Chol/PL mixing ratio used for preparation of lipid bilayer membranes modeling the lipid bilayer portion of animals of different lifespans. Symbols of the data for the mouse model (▲, Δ, ∇), for the pig model (■, □, ◇), and for human model (●, ○, ⊕). Filled symbols are for OTP values obtained with the CSL spin label. Because CSL cannot discriminate CBDs with the OTP, the values for the Chol/PL mixing ratios greater than 1 are a combination (averaged values of OTP) of those from the PL bilayer (with Chol) and from CBDs. Open symbols are for OTP values obtained with ASL spin label in PL bilayer surrounding CBDs (Δ, □, ○). Open symbols (∇, ◇, ⊕) show the OTP values in CBDs created at a Chol concentration greater than the appropriate Chol saturation limit.

The profiles of OTP (Figure 2A–C) do not contain data obtained with Chol-analog spin labels, ASL and CSL. These Chol analogs can be located in the PL bilayer part of the membrane and in the coexisting pure CBDs. However, only ASL, with its nitroxide fragment located in the membrane center and with the use of discrimination by the oxygen transport method [64], can discriminate CBDs. CSL, with its nitroxide fragment located in the polar headgroup region, can discriminate CBDs only with the use of the water-soluble relaxation agent NiEDDA [64]. We did not use this approach in the present study. An effort was made to prepare model membranes with Chol concentrations close to the Chol solubility limits at which CBDs were not yet present in the PL bilayer and model membranes oversaturated with Chol where CBDs were already formed. These allowed us to make a comparison of the profiles for membranes without Chol, saturated with Chol, and oversaturated with Chol. Measurements with ASL and CSL added values for the OTP sensed by these spin labels in the polar headgroup region and in the membrane center of PL bilayers. For these spin labels, we extended the measurements of the OTP values in all three models to the broad region of Chol content, from a Chol/PL mixing ratio of 0 up to a ratio of 3.3 (Figure 3). This allowed the determination of the Chol contents (Chol/PL mixing ratios) at which CBDs were present in all three membrane models. The data presented in Figure 3 complement those presented in membrane property profiles (Figure 1 and Figure 2) and clearly show that membrane properties (monitored here by the OTP) did not change in the PL bilayer portion in any of the three membrane models at the Chol saturation limit. The observed decrease in the OTP values measured in the CBDs was also expected. As we speculate in [23], smaller OTP values in the CBD can indicate a bigger size of these domains. It should be noted that the values of the OTP measured in CBDs in all three membrane models are very close in the whole region in which Chol content was measured. This suggests that the size of the CBDs depends on the Chol content in the membrane (as was suggested in [23]) and not on membrane PL composition.

The data presented in Figure 3 allowed us to carefully choose the Chol/PL mixing ratios for all three investigated models to ensure membrane saturation with Chol but not yet the formation of CBDs. These measurements were needed because model membranes were prepared using the film deposition method with artefactual Chol crystals formed during preparation (see [39,65,66] for more details). As follows from this figure, we used a Chol/PL mixing ratio slightly lower than 1.0 for the mouse model, slightly lower than 1.2 for the pig model, and slightly lower than 1.6 for the human model for membranes saturated with Chol. To ensure the presence of CBDs in the investigated membrane models, the Chol/PL mixing ratios were 2.1 for the mouse model, 2.4 for the pig model, and 3.2 for the human model.

### 3.4. Changes in Membrane Properties as a Function of Lifespan

**Figure 4:**
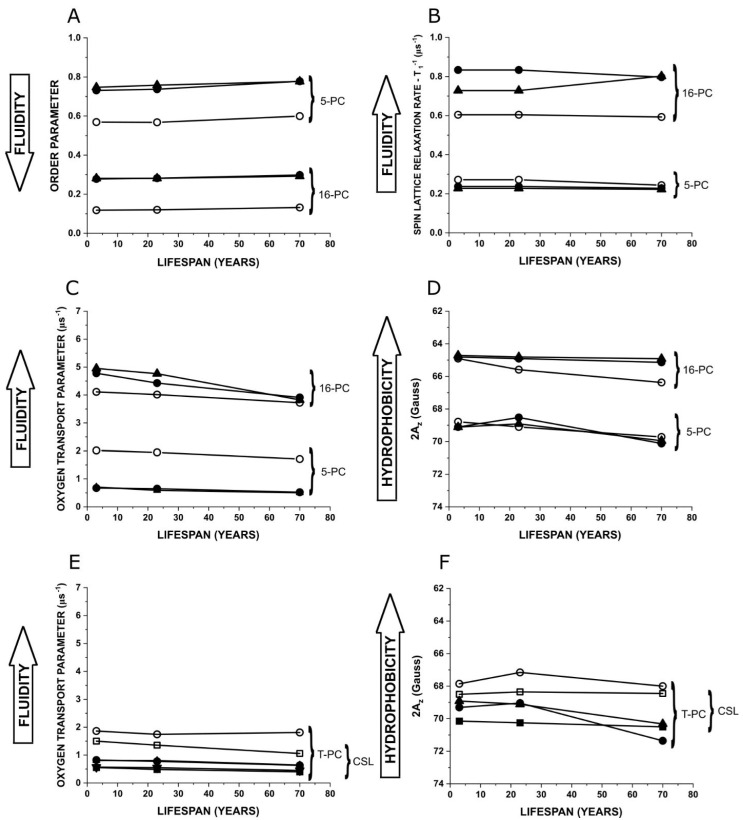
Changes in the physical properties of model membranes at selected positions (depths) in the membranes as a function of the lifespan of the chosen animal models. Symbols for 5-PC, 16-PC, and T-PC are the same as in Figure 1 and Figure 2. Because the results for CSL overlapped with those for T-PC, new symbols for CSL are employed [for lipid bilayers without Chol (□), bilayers with a Chol content at the saturation limit (■), and those with a Chol content well above the Chol saturation limit (▼)]. Values for hydrophobicity measured with CSL were taken from [67].

Figure 4 summarizes changes in the physical properties of model membranes as a function of the animal lifespans. For these comparisons, we chose the most sensitive positions across the membranes including the polar headgroup region (T-PC and CSL), the C5 position, where the rigid Chol structure is located in the membrane (5-PC), and the C16 position (16-PC), showing the properties in the membrane center. The first two figures (Figure 4A,B) show the changes in the properties of the PL acyl chains. Practically none of the parameters changed with the lifespan of the investigated animals, both in the absence of Chol and in the presence of saturating and oversaturating amounts of Chol (see the Discussion section for evaluated values of saturating and oversaturating amounts of Chol). The stability of the measured parameters is independent of the significant differences in the PL composition between membranes of the animal models. This is understandable and expected for membranes saturated and oversaturated with Chol, but for membranes without Chol, it is somewhat unexpected.

Figure 4C–F present the changes in the physical properties sensed by the nitroxide spin labels in their close vicinity. Measurements of the OTP were also possible in the polar headgroup region, and the data obtained with T-PC and CSL are included in Figure 4E. As shown in these figures, no changes in the polar headgroup region (T-PC and CSL positions, Figure 4E) or in the hydrocarbon region close to the membrane surface (5-PC position, Figure 4C) were observed in the membrane models of animals as a function of their lifespan, for membranes both in the absence and in the presence of Chol. However, the membrane center measurements with 16-PC showed a decrease in the OTP with the increase in the lifespan of animals for membranes saturated and oversaturated with Chol. For membranes without Chol, this decrease was negligible. Changes in the membrane hydrophobicity as a function of the lifespan of animals are more complex (Figure 4D,F). As expected, the hydrophobicity in the polar headgroup region (measured with T-PC and CSL, Figure 4F) significantly decreased in the presence of Chol in all membrane models. The value of the change is biggest for the human model. However, hydrophobicity in the hydrocarbon region of the membranes (measured with 5-PC, Figure 4D) was practically unaffected by the presence of Chol. The tendency of hydrophobicity in this membrane region to decrease with lifespan was noted. The hydrophobicity of the membrane center (measured with 16-PC, Figure 4D) was increased by the presence of Chol in the pig and human models but not in the mouse model. This change increased significantly with lifespan. It is the result of the stability of hydrophobicity for all three models for membranes saturated and oversaturated with Chol, and its significant decrease with lifespan for membranes without Chol can be explained by the increased concentration of SM in these models (15 mol% in the mouse model, 29 mol% in the pig model, and 66 mol% in the human model). The measured hydrophobicity parameter (values of 2*A*_Z_) can be compared among spin labels of the same kind (with the same structure of the nitroxide moiety) like n-PC. 2*A*_Z_ values for these spin labels can be transferred to the values of the dielectric constant (ε) using Figure 2 in [15]. The 2*A*_Z_ values obtained with T-PC and CSL show the changes and the extent of changes in hydrophobicity, which add significant information to the cross-membrane profiles of hydrophobicity.

### 3.5. Changes in the Chol Saturation Limit and the CST in Fiber Cell Membranes as a Function of Lifespan

**Figure 5:**
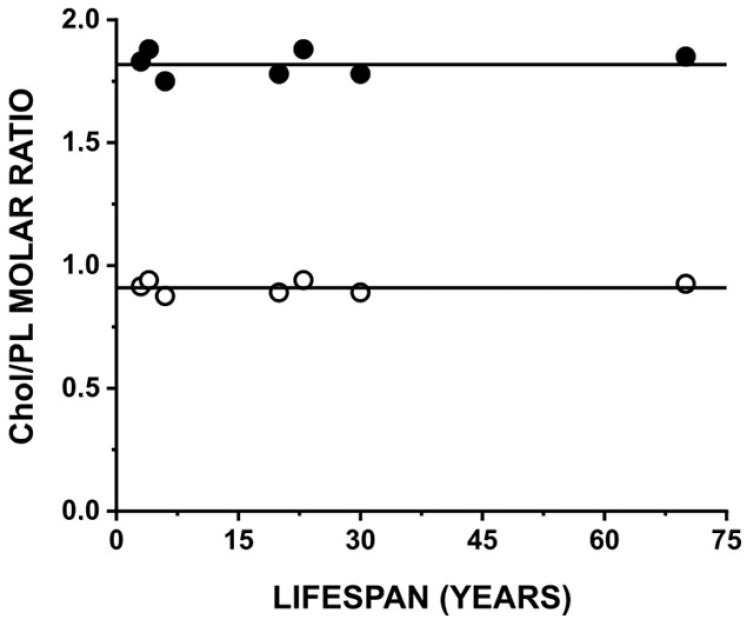
The relationship between the Chol saturation limit (○) and the CST (●) in the LLM and the maximum lifespans for the different species. CBDs should be formed at Chol concentrations greater than the Chol saturation limits. Chol crystals should be formed at Chol concentrations greater than the CSTs. Chol saturation limits and CSTs were evaluated based on the PL compositions taken from [4] and the phase diagram presented in [32]. Points are for mouse (3), rat (4), chick (6), sheep (20), pig (23), cow (30), and human (70) (maximum lifespan values are indicated in parentheses).

To clearly explain the mechanisms that keep fiber cell membrane properties so independent of the lifespans of animals, and independent of the drastic differences in their PL composition, we showed the relationship between the Chol saturation limit and the CST in LLMs and the maximum lifespans for the three investigated models (Figure 5). This figure also includes data for other animals with different lifespans. We based our calculations of Chol saturation limits and CSTs on the fluid phase diagram for mixtures of major lens PLs—namely, PC, PS, PE, and SM—with Chol, presented in [32]. This phase diagram was drawn based on the data obtained with the application of both EPR and DSC methods, which allowed us to obtain not only the appropriate values of the CSTs but also the values of the Chol saturation limits for the PC, PE, PS, and SM bilayers. The obtained values of the Chol saturation limit are 33 mol%, 50 mol%, 50 mol%, and 50 mol% Chol in the PE, PC, PS, and SM bilayers, respectively, and the obtained values of the CST are 50 mol%, 66 mol%, 66 mol%, and 66 mol% Chol in the PE, PC, PS, and SM bilayers, respectively [32]. The fluid phase diagram for mixtures of PS, PC, PE, SM, and Chol was drawn using these values, assuming that the Chol saturation limits and the CSTs for the PL mixture are the weighted sums of the Chol saturation limits and CSTs for individual PLs with a weight equal to the mole fraction of the individual PLs in the mixture. The same assumption was used to calculate the values of the Chol saturation limits and CSTs presented in Figure 5.

The data presented in Figure 5 confirm our conclusion (made based on profiles of physical membrane properties of the investigated models) that no differences in the organization and properties of membrane models of mice, pigs, and humans were detected. The evaluated Chol saturation limits and CSTs were the same for all animals with lifespans ranging from 3 to 70 years. This indicates that the Chol content above which CBDs and Chol crystals are formed is the same for all adult animals and is independent of the fiber cell membrane PL composition and the animal’s lifespan.

## 4. Discussion

We wanted to clearly understand the mechanisms that keep fiber cell membrane properties independent of the lifespans of animals, independent of the drastic differences in their PL composition. We expected this independence for adult animals containing saturating and oversaturating Chol contents because we have shown in many of our papers [20,21,22,23] that the presence of extremely high saturating and oversaturating Chol contents (presence of CBDs) in lipid bilayer membranes ensures the stability of their physical properties as well as the independence of the transmembrane profiles of these properties from changes in the membrane PL composition. This was confirmed for all three investigated models of animals with respective lifespans of 3, 23, and 70 years (see appropriate profiles in Figure 1 and Figure 2). All these mechanisms involve an extremely high Chol content. We can thus conclude that Chol is the key molecule in maintaining eye lens homeostasis.

As shown in Figure 5, the evaluated Chol saturation limits and CSTs were the same for all animal models, independent of the lifespans of the animals (ranging from 3 to 70 years) and of the drastic differences in their PL composition [4]. However, this seemingly unexpected result can be clearly explained. From the phase diagram [32], it follows that only PE can decrease the Chol saturation limits and CSTs. The PE contents in the PL compositions presented in [4] for adult animals of different lifespans are rather small, and they lie between 12 mol% and 25 mol%. No relationship between the PE content and the lifespan is indicated. This explains why the evaluated Chol saturation limits and CSTs are independent of animal lifespan and why their values are only a little scattered. The composition of other PLs differs drastically between animals of different lifespans. However, these differences cannot affect Chol saturation limits and CSTs for the investigated model membranes (mixture of different PL contents) because their individual Chol saturation limits and CSTs are the same. Thus, although the PL composition of the fiber cell membranes differs drastically between animals of different lifespans, it does not affect the Chol content at which CBDs and Chol crystals start to form. This was established for adult animals, where PL composition was already confirmed and Chol content was maximal.

The independence of the measured parameters of membrane physical properties between the membranes of animal models with different lifespans and different PL compositions is understandable and expected for membranes saturated and oversaturated with Chol. However, for membranes without Chol, this independence was somewhat unexpected because of the significant differences in the profiles of the physical membrane properties for the individual PLs forming these mixtures [10,15,17,18,32,54,61]. The lens fiber cells of animals with short lifespans possess membranes rich in glycerophospholipids with unsaturated acyl chains (for mice, 47% of PC, 17% of PE, and 17% of PS [4]) that are prone to lipid oxidation and subsequent lens opacification [4]. The fiber cell membranes of the mouse contain only 15% of sphingolipids [4]. For animals with long lifespans, fiber cell membrane composition is very different, with increased sphingolipids (in humans, 47% for dihydrosphingomyelin and 19% for sphingomyelin) and saturation of acyl chain content. These membranes become resistant to oxidation and are thus protected against lens opacification. Thus, changes in PL composition occurring in animals with longer lifespans ensure that the lenses of these animals can perform their function of focusing light on the retina throughout an animal’s life. The presented results of the independence of the physical properties of the fiber cell membrane of the animal models with different lifespans are provided with a new, significant meaning. Additionally, to protect membranes against lipid oxidation, these different PL compositions ensure that the structure, properties, and organization of lipids for all animals with different lifespans are constant, which also ensures proper functioning of the eye lenses. Thus, not only Chol but also an appropriate PL composition is a significant factor in maintaining the physical properties of the eye lens membrane at a constant level.

Finally, we recognize that the PL composition of the fiber cell membranes of animals with the shortest lifespan is very similar to those in the eye lenses of young humans (0 to 20 years old) [23]. Further comparisons allowed us to conclude that the changes in PL composition that occur in human fiber cell membranes during maturation and aging, to a certain degree, resemble those occurring with an increased animal lifespan [23]. In our recent paper [68], we proposed two major mechanisms developed during evolution to protect human eye lenses against age-related cataract development. One mechanism involved the remodeling of membrane PLs during the maturation of fiber cells to be resistant to oxidation (with increased sphingolipids and saturation of acyl chain content). The second protective mechanism involved increasing the lens membrane Chol content to saturation levels. The presented results indicate that, in addition to Chol, the effects of the PL composition on the structure, properties, and organization of lipids should be considered a significant factor in maintaining eye lens homeostasis.

## 5. Conclusions

The presented investigations allowed us to make three basic conclusions. First, the presence of high saturating and oversaturating Chol contents in fiber cell plasma membranes ensures the stability of the membranes’ organization and their physical properties being independent of the changes in the membrane PL composition that occur between different animal species. Thus, the same mechanism was developed during biological evolution to protect human [68] and other animals’ eye lenses against opacification. Second, no significant differences in the physical properties of membranes of any of the investigated models were detected for membranes without Chol. Thus, not only Chol but also an appropriate PL composition is a significant factor in maintaining eye lens homeostasis. Third, the changes in the PL composition in fiber cell membranes occurring with the increase in animal lifespan, from 3 to 70 years, resemble those occurring in human fiber cell membranes during maturation and aging. Thus, the obtained data can be used to explain the mechanisms protecting human eye lenses against opacification during aging.

## Data Availability

The original contributions presented in this study are included in the article/Supplementary Material. Further inquiries can be directed to the corresponding authors.

## Supplementary Materials

The following supporting information can be downloaded at https://www.mdpi.com/article/10.3390/biom15060851/s1. References [15,46,59,60,64,69] are cited in the Supplementary Materials.

## Abbreviations

The following abbreviations are used in this manuscript:

ASL	Androstane spin label
CBD	Cholesterol bilayer domain
Chol	Cholesterol
CSL	Cholestane spin label
CST	Cholesterol solubility threshold
DHSM	Dihydrosphingomyelin
EPR	Electron paramagnetic resonance
LLM	Lens lipid membrane
n-PC	1-Palmitoyl-2-(n-doxylstearoyl)phosphatidylcholine
O	Oleic
OTP	Oxygen transport parameter
P	Palmitic
PC	Phosphatidylcholine
PE	Phosphatidylethanolamine
PL	Phospholipid
PS	Phosphatidylserine
SM	Sphingomyelin
SR	Saturation recovery
*T*-PC	Tempocholine-1-palmitoyl-2-oleoylphosphatidic acid ester
TPX	Methylpentene polymer
T1	Relaxation time
9-SASL	Ninedoxylstearic acid spin label
