# Supplementary material for: Physical Properties of Lens Membranes in Animals with Different Lifespans
Source: Biomolecules. 2025 Jun 10;15(6):851. doi: 10.3390/biom15060851 (PMC12190193; doi:10.3390/biom15060851)
Supplement: Supplementary file 1 [file biomolecules-15-00851-s001.zip › biomolecules-3612458-supplementary.pdf]

Supplementary Materials

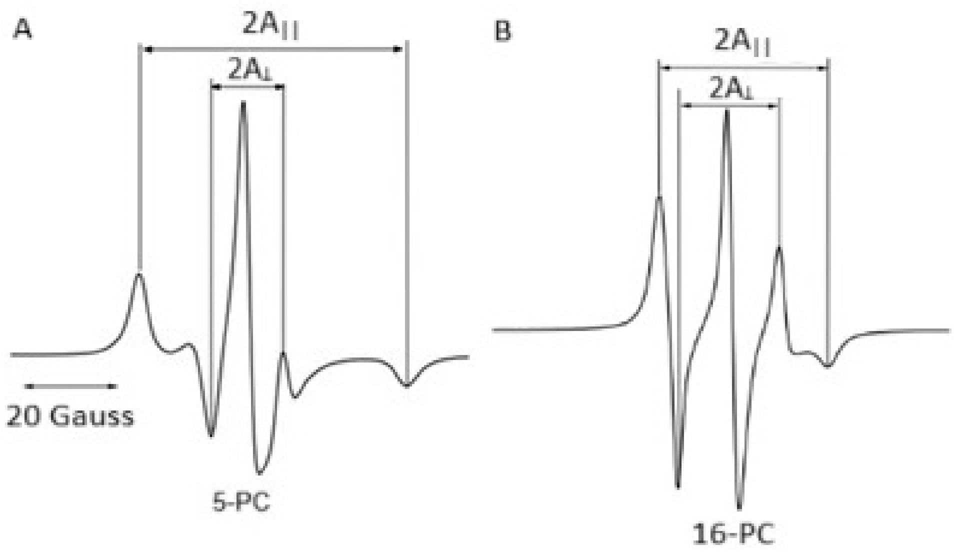

Figure S1. Representative CW EPR spectra recorded at 37°C for the deoxygenated human membrane model (this composition included 11 mol% of PC, 15 mol% of PE, 15 mol% of PS, and 66 mol% of SM) saturated by cholesterol with 5-PC (A) and 16-PC (B). Order parameter (amplitude of the wobbling of the acyl chain segment) was calculated using the equation $S = 0.5407(A_{\|}-A_{\perp})/a_0$, where $a_0=(A_{\|}-A_{\perp})/3$. Measured values for evaluating order parameters are indicated (for more details see the reference [S1]).

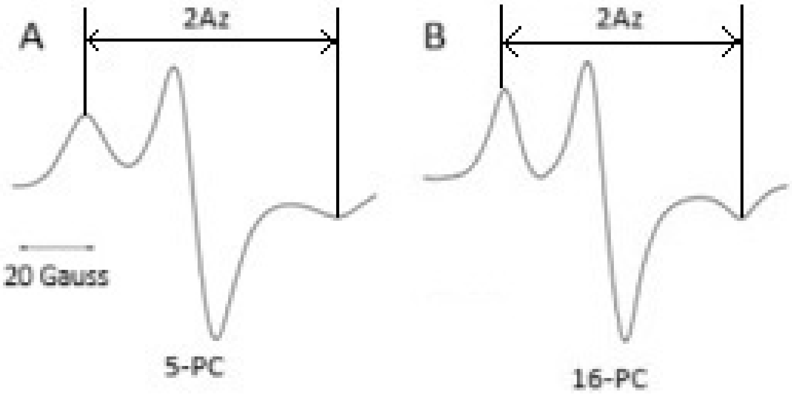

Figure S2. Representative CW EPR spectra for 5-PC (A) and 16-PC from for the deoxygenated human membrane model saturated by cholesterol, recorded at −165°C to cancel motional effects. Hydrophobicity was measured as the hyperfine interaction. See also reference [S2] for more detailed explanation.

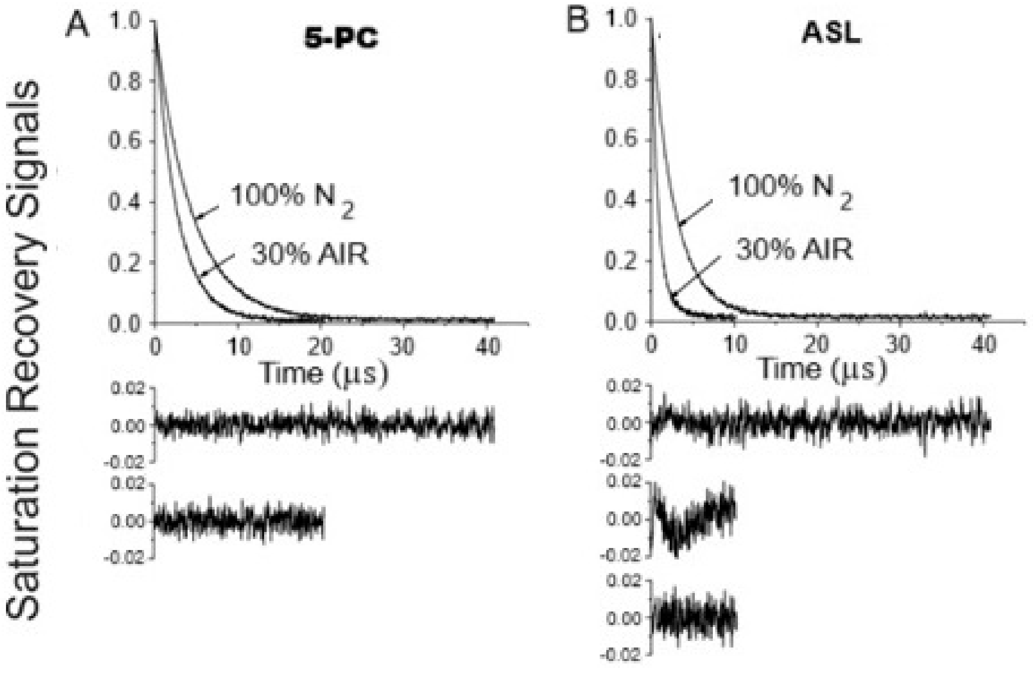

Figure S3. Representative SR EPR signals with fitted curves recorded at 37°C for 5-PC (A) and ASL (B) spin labels from for the deoxygenated (equilibrated with a 100% N_2) and equilibrated with the gas mixture of 30% air/70% N_2 for human membrane model saturated by cholesterol. All SR EPR signals from deoxygenated samples were satisfactory fitted to a single exponential function (see residuals) giving spin-lattice time, T_1, values and spin lattice relaxation rates, T_1^{-1}, values. Fittings were performed using single-exponential equation:

$$I(t) = I_o \exp-(tT_1^{-1}), \qquad (S1)$$

where I(t) and I_o are, respectively, amplitudes of SR signals at time t and immediately after the end of the saturating microwave pulse. T_1 is the spin-lattice relaxation time. T_1 values (indicated in Figure S3A for 5-PC) were used as a conventional way to assess membrane fluidity, more correctly, rotational diffusion rate of the acyl chain fragment to which the nitroxide moiety is rigidly attached. In deoxygenated samples T_1^{-1}s, depend on the rate of the spin label rotational diffusion [S3,S4], and with the use of the appropriate calibration curves, can be transferred to rates of rotational diffusion coefficients [S5].

Signals for ASL from samples equilibrated with a gas mixture of 30% air/70% N_2 (Figure S3B) can be satisfactorily fitted to the double exponential function (residual for single exponential fit (second residual) is not satisfactory and the residual for double exponential fit (third residual) is satisfactory. Fittings to the double-exponential functions were performed using equation (S2):

$$I(t) = I_{o1} \exp-(tT_{11}^{-1}) + I_{o2} \exp-(tT_{12}^{-1}), \qquad (S2)$$

where the subscripts 1 and 2 indicate parameters coming from two different lipid environments. Detailed description of the process for the discrimination of CBDs is done in [S6].

References

S1. Marsh, D. Electron Spin Resonance: Spin Labels. In Membrane spectroscopy; Grell, E., Ed.; Springer-Verlag: Berlin, 1981; pp. 51–142.

S2. Subczynski WK, Wisniewska A, Yin JJ, Hyde JS, Kusumi A. Hydrophobic barriers of lipid bilayer membranes formed by reduction of water penetration by alkyl chain unsaturation and cholesterol. Biochemistry. 1994; 33:7670–7681.

S3. Marsh, D. Molecular Order and T1-Relaxation, Cross-Relaxation in Nitroxide Spin Labels. J Magn Reson 2018, 290, 38–45.

S4. Robinson, B.H.; Haas, D.A.; Mailer, C. Molecular Dynamics in Liquids: Spin-Lattice Relaxation of Nitroxide Spin Labels. Science. **1994**, 263, 490–493.

S5. Subczynski WK, Widomska J. Spin-Lattice Relaxation Rates of Lipid Spin Labels as a Measure of Their Rotational Diffusion Rates in Lipid Bilayer Membranes. Membranes (Basel). Sep 30 2022; 12(10): 962

S6. Raguz, M.; Mainali, L.; Widomska, J.; Subczynski, W.K. Using Spin-Label Electron Paramagnetic Resonance (EPR) to Discriminate and Characterize the Cholesterol Bilayer Domain. Chem. Phys. Lipids 2011, 164, 819–829.
